# On the Elimination of Mercury from the System

**Published:** 1862-02

**Authors:** 


### ON THE ELIMINATION OF MERCURY FROM THE SYSTEM.

Dr. Schneider has recently made some researches on the elimination of mercury during and after a mercurial treatment, which have given the following results:—During the internal administration of such preparations, the electrolytic examination of the urine shows always traces of mercury; but the quantities are so small that the amount of urine discharged within twenty-four hours is often not sufficient for a satisfactory result, and the metal must be searched for in the whole of the urine discharged within three to six days. Traces of mercury were also found in the urine of a patient who had not taken any internally, but had been treated with mercurial ointment. The elimination of the drug by the urine lasts for some time after the mercurial ointment has been discontinued. In the first week afterward it is invariably found, and in two cases it was even discovered in the fourth and the sixth week afterwards. After six weeks, Dr. Schneider has never succeeded in discovering traces of mercury by electrolysis. The elimination of it is not promoted by the administration of iodide of potassium, as the quantity of mercury contained in the urine was not at all increased, but rather diminished, when that drug was given; and if it was taken a few months after the end of the mercurial treatment, mercury did not reappear in the system, probably because nothing was left behind. One patient, who had suffered with mercury (the last time very energetically, having used twenty drachms of ointment, equal to more than 120 grains of mercury), died of pericarditis two months after the third course, and Dr. Schneider could therefore search for mercury in the internal organs. The result was that the bones, the brain, and the spleen, which are generally believed to retain mercury for years, did not contain a trace of it, while the kidneys showed an infinitesimal quantity of the metal, the result of the examination of the liver being doubtful. It is, therefore, evident that mercury entirely disappears from the system a short time after the end of treatment, and cannot, therefore, cause secondary syphilitic symptoms; nor can it be eliminated from the system years after the treatment by Dr. Caplin's electro-chemical baths. In two cases of hydrargyrosis, considerable quantities of mercury were discovered in the urine, and in the one of them, which ended fatally, also in the internal organs, especially in the liver. Urine which contains mercury does not necessarily contain albumen also; but in the two last-mentioned cases of hydrargyrosis there was albumen in the urine. The saliva which is collected during a mercurial treatment does not contain any mercury. The quantity of mercury which is eliminated after the end of the treatment amounts to about one fourth of that actually administered.—*Boston Med. Journal.*

